# Disaster resilience in tertiary hospitals: a cross-sectional survey in Shandong Province, China

**DOI:** 10.1186/1472-6963-14-135

**Published:** 2014-03-25

**Authors:** Shuang Zhong, Xiang-Yu Hou, Michele Clark, Yu-Li Zang, Lu Wang, Ling-Zhong Xu, Gerard FitzGerald

**Affiliations:** 1 Center for Emergency & Disaster Management, School of Public Health and Social Work & Institute of Health and Biomedical Innovation, Queensland University of Technology, Brisbane, QLD, 4059, Australia; 2 School of Clinical Sciences, Queensland University of Technology, Brisbane, QLD, 4059, Australia; 3 School of Nursing, Shandong University, #44 Wenhua West Road, Jinan, Shandong Province, 250012, P. R. China; 4 Health Department of Shandong Province, Jinan, Shandong Province, 250014, P. R. China; 5 School of Public Health, Shandong University, #44 Wenhua West Road, Jinan, Shandong Province, 250012, P. R. China

**Keywords:** China, Current status, Disaster management, Emergency, Evaluation framework, Hospital resilience

## Abstract

**Background:**

Hospital disaster resilience can be defined as a hospital’s ability to resist, absorb, and respond to the shock of disasters while maintaining critical functions, and then to recover to its original state or adapt to a new one. This study aims to explore the status of resilience among tertiary hospitals in Shandong Province, China.

**Methods:**

A stratified random sample (n = 50) was derived from tertiary A, tertiary B, and tertiary C hospitals in Shandong Province, and was surveyed by questionnaire. Data on hospital characteristics and 8 key domains of hospital resilience were collected and analysed. Variables were binary, and analysed using descriptive statistics such as frequencies.

**Results:**

A response rate of 82% (n = 41) was attained. Factor analysis identified four key factors from eight domains which appear to reflect the overall level of disaster resilience. These were hospital safety, disaster management mechanisms, disaster resources and disaster medical care capability. The survey demonstrated that in regard to hospital safety, 93% had syndromic surveillance systems for infectious diseases and 68% had evaluated their safety standards. In regard to disaster management mechanisms, all had general plans, while only 20% had specific plans for individual hazards. 49% had a public communication protocol and 43.9% attended the local coordination meetings. In regard to disaster resources, 75.6% and 87.5% stockpiled emergency drugs and materials respectively, while less than a third (30%) had a signed Memorandum of Understanding with other hospitals to share these resources. Finally in regard to medical care, 66% could dispatch an on-site medical rescue team, but only 5% had a ‘portable hospital’ function and 36.6% and 12% of the hospitals could surge their beds and staff capacity respectively. The average beds surge capacity within 1 day was 13%.

**Conclusions:**

This study validated the broad utility of a framework for understanding and measuring the level of hospital resilience. The survey demonstrated considerable variability in disaster resilience arrangements of tertiary hospitals in Shandong province, and the difference between tertiary A hospitals and tertiary B hospitals was also identified in essential areas.

## Background

Since the outbreak of severe acute respiratory syndrome (SARS) in 2003 and the Wenchuan earthquake in 2008, substantial resources have been devoted to improving disaster resilience in China, with a particular emphasis on mitigating the impact of wide-spread infectious diseases and mass casualty incidents [1,2]. Adequate progress can only be achieved by integrating local, provincial, and national systems [3]. Health systems are essential to enhance disaster resilience, and therefore planning at all levels should include health care facilities, such as tertiary hospitals, primary health care facilities, public health departments, and emergency medical services [4]. Within regional health systems, tertiary hospitals are the key component, as they are the main providers of health care during disasters. They also provide leadership during response phase of a disaster, and represent a critical linkage for disaster management for the whole system.

During disasters, hospitals need to withstand the event, whilst being able to maintain and surge their medical capacity, in order to respond to sudden and significant increases in health demand [5-7]. Resilience is an emerging concept that has recently been added to the disaster management context, which describes this ability [8-10]. The resilience of hospitals can be defined as their ability to resist, absorb, and respond to the shock of disasters while maintaining critical functionality, and then to recover to their original state or adapt to a new one [8-12].

China is afflicted by many kinds of disasters, including natural disasters, manmade disasters and pandemics. Its population size amplifies the impact of these disasters on health and wellbeing. To date, few studies have evaluated the level of hospital disaster preparedness and management arrangement in China [13-16]. Moreover, standardised or consistent methods for describing and measuring hospital resilience or relevant comprehensive ability are lacking [17]. Understanding the status of hospital disaster resilience is the first step in planning to enhance effective emergency response of hospitals. This article aims to explore the current status of disaster resilience in tertiary hospitals in Shandong Province. It has four objectives: (1) to identify the current status of tertiary hospitals’ ability to cope with disasters in the Province; (2) offer references for similar studies; (3) to test the construct validity and the utility of an emerging framework as a basis for understanding and measuring hospital disaster resilience; and (4) to identify any variability in hospital disaster resilience in Shandong Province using this framework, in order to inform a more cohesive approach to health authorities and hospital managers.

## Methods

The survey questionnaire used in this study was developed from an established framework of hospital disaster resilience. The framework was derived from analysis of existing literature and through a three-round of Modified-Delphi consultation with key experts in China [18]. The resultant questionnaire consists of 9 sections and 166 items (the framework and questionnaire can be found in the Additional files 1 and 2). Most questions are in the format of binary variables, and can be answered by “yes” or “no”. The relevant framework and questionnaire can be accessed from the additional files.

The feasibility and suitability of the questionnaire were tested by a pilot study of three hospitals (n = 3). For the purpose of this study, we collected and analyzed the data focused on the following areas of interest: (1) hospital demographic data; (2) hospital internal safety (e.g., infrastructural safety and strategies for infectious diseases); (3) emergency leadership and cooperation; (4) disaster plan; (5) disaster stockpile and logistics management; (6) emergency staff; (7) emergency critical care capability (e.g., on-site rescue, hospital treatment, surge capacity); (8) training and drills; (9) and disaster recovery mechanism. Excluding the first section of the survey which addressed demographic information, Sections 2–9 (covering 161 survey questions) represent the eight key domains of the established evaluation framework of hospital disaster resilience.

A tertiary hospital is defined as cross-regional facility providing comprehensive and specialized medical care. In China they are further classified into subgroups: Grade A, Grade B, and Grade C according to their service levels, size, medical technology, medical equipment, management and medical quality [19]. Shandong province is the second largest province and is located in the east of China. In this study, a cross-sectional survey was conducted in tertiary hospitals of Shandong province in China. A total of 50 tertiary hospitals in Shandong Province were selected using stratified random sampling according to their subgroups (i.e., Grade A, B, C). The sample was composed of 28 tertiary A hospitals, 20 tertiary B hospitals and 2 tertiary C hospitals, which was selected using the contact list obtained from the Provincial health department. Between January 2013 and June 2013, the questionnaire accompanied by an official letter from the provincial health department stating the importance of the survey was sent to these hospitals. Each hospital was asked to designate a department director to be responsible for coordinating the completion of the questionnaire. Ethical approval was obtained from Queensland University of Technology (approval number 1200000170) and written informed consent obtained from each participant hospital.

Each returned questionnaire was reviewed for its completeness and consistency. For those questionnaires which were incomplete and/or contained inconsistent responses follow-up telephone calls were made to ensure completeness and consistency. The data from returned questionnaires were then transferred into a database, which was set up using Microsoft office access 2007. Data was checked, cleaned, and analysed using SPSS Statistics version 21. As the study was conducted in China, the Chinese language was used to capture responses, but the results subsequently were translated into English for final analysis and reporting.

A score was assigned for the binary variables (e.g., “is there”). Two options of “yes” or “no” are assigned to the score of “1”or “0” respectively. Then, the scores of each domain were calculated by adding together the score of all the relevant questions. A total score was calculated by summing the score across all eight domains, which is a proxy for measuring disaster resilience in an institution. The higher the total score, the better the hospital’s disaster resilience.

Further analyses were conducted to understand the correlation between resilience domains and the descriptive information about the hospitals. A mean score and ninety-five percent confidence interval of means (95% CI) were used to describe each resilience domain. Comparisons of the mean score of each resilience domain among different hospital categorizes were performed, with p < 0.05 as the level of statistical significance. Due to the small sample, non-parameter test (Mann–Whitney Test) was used as the statistical method. Factor Analysis was used to test the construct validity of the evaluation framework by extracting key factors of disaster resilience from the different domains. Most variables in this study were analysed using descriptive statistics such as frequencies and percentage.

## Results

A response rate of 82% (n = 41) was attained. After analysis of the data from these 41 hospitals, it was found that the eight domains of disaster resilience have good overall internal statistical consistency (Cronbach alpha = 0.780). A comparison of these domains among different categories of hospitals is shown in Table 1. The mean score of each domain of tertiary grade A hospitals was higher than that of tertiary grade B separately, and statistical difference was confirmed among most domains. The mean score of general hospitals was higher than that of specialized hospitals in most domains. However, only the statistical difference among two domains was tested. The mean score of hospitals that were assigned the mission of regional disaster rescue was higher than those hospitals without this mission. Statistical difference among most domains was tested. In addition, most of the hospitals (92.3%) that has been assigned missions were tertiary A hospitals in the sample.

**Table 1:** Comparison of eight domains of hospital resilience, categorized by different characteristics of hospitals, Shandong, China, 2012

**Var.**		**No.**	**Domains**							
			**Leadership**	**Plan**	**Stockpile**	**Safety**	**Critical care**	**Staff**	**Training**	**Recovery**
			**Mean 95% CI**	**Mean 95% CI**	**Mean 95% CI**	**Mean 95% CI**	**Mean 95% CI**	**Mean 95% CI**	**Mean 95% CI**	**Mean 95% CI**
Level	Tertiary A	27	5.78	6.22	2.26	8.07	9.34	10.59	8.98	0.89
			5.35,6.21^*^	5.81,6.64^*^	1.98,2.54^*^	7.44,8.70^*^	*7.53,11.16*	*8.16,13.02*	7.62,10.33^*^	0.501.27^*^
	Tertiary B	14	4.43	3.36	1.29	6.93	8.71	10.29	5.32	0.07
			3.89,4.97^*^	2.49,4.22^*^	0.63,1.94^*^	6.31,7.55^*^	*5.74,11.69*	*6.26,14.31*	3.56,7.08^*^	−0.08,0.23^*^
Type	General	27	5.52	5.11	2.00	7.63	10.49	12.77	8.27	0.67
			*5.05,5.99*	*4.33,5.90*	1.59,2.41^*^	*7.09,8.17*	8.59,12.39^*^	10.25,15.30^*^	6.65,9.89^*^	*0.27,1.06*
	Specialized	14	4.93	5.50	1.79	7.79	6.50	6.07	6.68	0.50
			*4.23,5.63*	4.63,*6.37*	1.27,2.30^*^	6.72,*8.85*	4.55,8.45^*^	4.18,7.96^*^	5.17,8.20^*^	*0.12,0.88*
Disaster	Assigned	13	6.23	6.46	2.38	8.23	11.77	14.23	11.06	1.23
Mission			5.57,6.89^*^	5.62,7.30^*^	*2.08,2.69*	*7.48,8.98*	9.29,14.25^*^	10.58,17.88^*^	9.43,12.69^*^	0.67,1.79^*^
	No mission	28	4.89	4.68	1.71	7.43	7.90	8.74	6.18	0.32
			4.49,5.29^*^	4.00,5.35^*^	*1.29,2.13*	*6.81,8.05*	6.14,9.67^*^	6.49,10.99^*^	5.00,7.36^*^	0.04,0.60^*^
Total		41	5.32	5.24	1.93,	7.68	9.13	10.48	7.73	0.61

Emergency leadership and cooperation, (highest score = 7); disaster plan, (highest score = 7); disaster stockpile and logistics management, (highest score = 4); hospital safety, (highest score = 9); emergency critical care capability, (highest score = 19); emergency staff, (highest score = 17); trainings and drills (highest score = 15); recovery mechanism (highest score =3).

*P < 0.05; Tested by non-parameter test (Mann–Whitney Test).

*MS*, Mean score.

95% CI: 95% confidence interval of means.

Factor Analysis (using Initial Component Matrix, and Rotated Component Matrix by Varimax with Kaiser Normalization) was chosen to extract key factors of disaster resilience from the eight different domains [20]. After analysis, a four factor solution was identified that can be used to represent all the domains. The score of each factor was obtained by regression analysis applied to the sample. According to Table 2, each factor score of the hospital sample can be expressed using the following modelling:

$$\begin{array}{l} F_{1}=0.524X_{1}+0.570X_{2}\\ \;+0.191X_{3}‒0.080X_{4}‒0.220X_{5}‒0.083X_{6}‒0.147X_{7}\\ \;+0.080X_{8} \end{array}$$

$$\begin{array}{l} F_{2}=0.030\;X_{1}‒0.315\;X_{2}+0.138\;X_{3}+0.443\;X_{4}\\ \;+0.182\;X_{5}+0.659\;X_{6}‒0.214\;X_{7}‒0.211X_{8} \end{array}$$

$$\begin{array}{l} F_{3}=‒0.253\;X_{1}‒0.116\;X_{2}+0.093\;X_{3}+0.007\;X_{4}\\ \;+0.364\;X_{5}‒0.318\;X_{6}+0.936\;X_{7}‒0.199X_{8} \end{array}$$

$$\begin{array}{l} F_{4}=‒0.144\;X_{1}+0.288\;X_{2}‒0.109\;X_{3}‒0.119\;X_{4}\\ \;+0.148\;X_{5}‒0.167\;X_{6}‒0.148\;X_{7}+0.972X_{8}. \end{array}$$

**Table 2:** Component score coefficient matrix

**Domains**	**Component factors**			
	**F1**	**F2**	**F3**	**F4**
1. Emergency critical care (X_1_)	.524	.030	–.253	–.144
2. Emergency staff (X_2_)	.570	–.315	–.116	.288
3. Emergency training and drills (X_3_)	.191	.138	.093	–.109
4. Crisis leadership and cooperation (X_4_)	–.080	.443	.007	–.119
5. Disaster plans (X_5_)	–.220	.182	.364	.148
6. Recovery (X_6_)	–.083	.659	–.318	–.167
7. Disaster stockpiles and logistics (X_7_)	–.147	–.214	.936	–.148
8. Hospital internal safety (X_8_)	.080	–.211	–.199	.972

Extraction Method: Principal Component Analysis.

Rotation Method: Varimax with Kaiser Normalization.

The four factors were identified and named as: hospital disaster medical care capability (F_1_), hospital disaster management mechanism (F_2_), hospital disaster resources (F_3_) and hospital safety (F_4_). These four factors account comprehensively for the overall level of disaster resilience capability. The weight of each factor was assigned using the total variance from factor analysis (the weight of each factor is assigned by the proportion of the variance contribution of each principal to the cumulative variance contribution of the four primary factors) [20]. Then the evaluation model of hospital disaster resilience can be established, and the overall level of hospital disaster resilience (F) can be calculated using the model as below:

$$F=0.615F_{1}+0.202F_{2}+0.103F_{3}+0.080F_{4}.$$

The overall score of disaster resilience (F) for each hospital of this study can be calculated accordingly with their relative rank are listed as Table 3. Due to ethical issues, the hospital name is replaced with hospital ID in this study. According to Table 2, there are 20 hospitals whose average of comprehensive scores (F) were positive, which account for about 50% of all of the sample quantity. It illustrated that the hospital disaster resilience in the province was still insufficient with a big difference among those sampled. Similarly, the score of each four factors (F_1_, F_2_, F_3_, and F_4_) can be calculated and ranked respectively according to Table 2, and thus to identify the score of factors which tend to be relatively lower in an area and should probably be the highest priority for strengthening resilience. As a result, it was found that in the study sample, the score of hospital disaster management mechanism (F_2_) was relatively lower than the other three factors with less than 50% of hospitals having positive scores. Below we describe in detail of the status of each of these four factors in tertiary hospitals of the study province.

**Table 3:** Overall score of hospital disaster resilience (F) and its rank in the hospital sample

**ID**	**F**	**Rank**	**ID**	**F**	**Rank**	**ID**	**F**	**Rank**	**ID**	**F**	**Rank**
1	−0.554	32	11	−0.33	31	21	0.7885	5	31	−0.235	28
2	−0.997	37	12	0.482	11	22	0.3393	15	32	0.412	12
3	0.384	13	13	−0.195	26	23	1.120	1	33	0.0990	18
4	−0.206	27	14	−0.970	36	24	−0.093	23	34	0.733	7
5	−0.750	35	15	−1.189	41	25	0.095	19	35	0.331	16
6	−0.147	24	16	−1.126	40	26	−0.262	30	36	0.733	8
7	0.049	20	17	−1.005	38	27	−0.085	22	37	1.088	2
8	−0.239	29	18	−1.116	39	28	0.774	6	38	0.932	3
9	0.262	17	19	−0.04	21	29	0.623	9	39	−0.176	25
10	0.550	10	20	0.8624	4	30	−0.572	33	40	0.360	14
									41	−0.729	34

### Hospital safety (Factor 1)

Most of the responding hospitals (92.7%) had developed syndromic surveillance systems for certain high risk of infectious diseases (e.g., SARS, Human H_5_N_1_ Avian Flu, and Human H_9_N_11_ Avian Flu) and required that physicians on duty report any suspicious cases to the hospitals’ presidents. 92.7% of responders had established a direct online reporting system to report suspicious cases and shared data with the local health authority. Most of responders (85.4%) could analysis surveillance data regularly.

Only 12.2% of all the responding hospitals had local risk evaluation for hospital prevention and mitigation, and all of them are tertiary A hospitals. More than half (68.3%) had evaluated critical infrastructure vulnerability and safety standards. Yet, this percentage varied with 88.9% within tertiary A hospitals but only 28.6% within tertiary B hospitals. All the hospitals had considered construction safety standards for the risk of fire, and most (92.7%) had considered using isolated pathways and designated areas for infectious diseases. Comparatively, a relatively lower percentage had considered building to a higher standard or level of resistance than the local criteria for earthquake disasters (82.9%), and floods (73.2%). Almost all of them (97.6%) had strategies to evacuate and protect existing patients when the hospital is at risk, but only 61.0% reported to have alternative emergency energy and facilities for backup (including power, water, oxygen and telecommunication). The percentage having alternative backup was found different among tertiary A and tertiary B hospitals, with 74.1% and 35.7% respectively.

### Hospital disaster management mechanism (Factor 2)

All of the surveyed hospitals (100%) had a general plan for public emergencies. While only a small percentage (19.5%) had specific plans based on the specific requirements of a single hazard, this was 75% in tertiary A hospitals. The most common hazards identified in the specific plans are infectious diseases (92.7%), internal medical accidents (90.2%), and public health emergencies, such as occupational or food poisoning (73.2%). Only a small percentage had established specific plans of dealing with natural disasters, such as fire (68.3%), earthquakes (48.8%), floods (36.6%), and even less for bio-terrorism and nuclear terrorism (31.7%). Regarding standard operating procedures, over four-fifths of the responding hospitals (85.4%) possessed a protocol to initiate the plan, so as to guarantee the availability of staff, equipment and resources, while less than three-quarters (73.2%) had a classification response system for different levels and different phases of events. Most hospitals (87.8%) reported that they could operate in accordance with the plan during disasters. Approximately half of them (51.2%) had evaluated and revised their emergency plan at least once in the last two years, and a similar percentage (53.7%) reported the content of plans was disseminated by key staff through regular meetings or training.

Regarding the incident command system, all reported to have a command center and most (97.6%) had designated a specific department to be responsible for the relevant work. As for communication, 87.8% had coordinating meeting during emergencies with key staff from different hospital departments, and less (48.8%) had a public and mass media communication protocol, and less still (43.9%) had attended the local coordinating meeting with other emergency departments, such as CDC (Center for Disease Prevention and Control), pre-hospital emergency system, healthcare facilities, blood and resource center, and local government. It was noteworthy that general hospitals did not appear to be obviously superior to specialized hospitals in disaster planning or cooperation with other facilities. However, the hospitals with assigned rescue missions had better cooperation mechanisms, among them with 61.5% attended the local coordinating meeting, while a smaller percentage (42.9%) in other hospitals.

Less than half (48.8%) had after-event evaluation report to capture lessons to be learned, and to assist to evaluate hospital vulnerability, and adapt strategies for improving future performance. However, a larger percentage (59.3%) of tertiary A hospitals had this evaluation report compared with tertiary B hospitals (28.6%). Only 26.8% had specific channels of investing money, transferring staff, and purchasing equipment for recovery. Approximately one-fifth (19.5%) of hospitals indicated that they were involved or would be involved in the health related work of affected communities (e.g.., rehabilitation and psychological consultation, health evaluation and health intervention of the community).

### Hospital disaster resources (Factor 3)

Our results revealed that 75.6% of the participating hospitals had stockpiles of emergency drugs, and 43.9% had signed contracts with emergency drug-supplies to provide drugs during emergencies, only 22.0% had signed Memorandum of Understandings (MOUs) with other hospitals to share emergency drugs during emergencies. 48.8% had drug-distribution plans to identify distribution priority of drugs during crisis, and 41.5% could be able to load and deliver emergency drugs for on-site rescue. With regards to other medical materials, 87.5% had stockpiles of emergency materials (e.g., food, water, stretcher, and tourniquet). But only 29.3% could share and obtain these materials from other hospitals during emergencies. It was noteworthy that a greater percentage of tertiary A hospitals had a signed Memorandum of Understandings (MOUs) to share emergency materials than tertiary B hospitals (42.9% among tertiary A and 22.2% among tertiary B hospitals). To protect staff, some of the hospitals had biohazard protective suits (58.5%), goggles (63.4%), ventilator (73.2%), and N95 Masks (41.5%). Only 24.4% had purchased all the mentioned personal protective equipment (PPE). There was also a greater percentage of tertiary A hospitals had access to all PPE than tertiary B hospitals (33.3% among tertiary A and 7.1% among tertiary B).

### Hospital disaster medical care capability (Factor 4)

The most important function of hospitals during emergencies is maintaining and surging services to ensure medical care for victims of disasters, either on-site or within hospitals. For onsite-rescue, most (92.7%) had their own ambulances, among them 73.7% had ward-type ambulances, but only 15.8% had access to a negative pressure isolation ambulance. Only two of them had their own rescue helicopters and access to a helicopter landing pad. More than half (65.8%) could dispatch emergency staff during disasters for the on-site rescue. About one third (31.7%) could organise an independent rescue team that is equipped with emergency package of supplies for living 3 days (including daily necessities, a set of emergency package, and first aid kit, et al.). Not surprisingly, among them most (92.3%) were tertiary A hospitals. Less than a quarter of the hospitals (22.0%) were equipped with portable medical equipment (e.g., portable breathing machine, ECG monitoring machine, and the X-ray machine), and all of these hospitals were tertiary A. The rescue teams comprised physicians, nurses and administrative staff from various departments (e.g., surgery department, medicine department, psychology department, infection control department and management department), and the dispatched staff number had a wide range from 13 persons to 103 persons. Over a third (36.6%) of the responding hospitals had an on-site command vehicle and a similar proportion (39.0%) had on-site communication equipment for data transmission, video-audio connection, and remote consultation. However, only 4.88% hospitals had a ‘portable hospital’ or the capability to support field surgery, and other critical care in the field, which is similar to the function of ICU (using vehicles which are equipped with beds, and portable medical equipment).

For hospital treatment, 90.2% had emergency beds, 80.5% had isolation beds, and 65.9% had Intensive Care beds. In terms of capacity to treat patients with different medical needs or case-mix 73.2% had orthopaedic beds, 56.1% had special beds for burns (e.g., suspension bed, emancipated beds) and 59.5% had hyperbaric oxygen chamber. While 73.2% had achieved capacity (e.g., space, beds and experts) for treating mass casualty of trauma, 73.2% for infectious diseases, 48.8% for mass casualty of blast injury, gunshot wounds and crush injury, 46.3% for acute chemical poisoning, only 17.1% could treat radiation issues. In this study mass casualty capacity refers to each hospital is to assess itself on its capacity to accept at least 30 patients of the same disease within a short period [21]. Most of the responding hospitals had medical care equipment, such as breathing machine (100%), vital signs monitors (100%), defibrillator machines (90.2%), and cardiac resuscitation devices (70.7%).

For surge capacity, 65.9% had prepared conditions (e.g., electricity, oxygen, water and space) to surge patient-care beds, while 53.7% had concrete surging plans, and among them 66.7% within tertiary A but only 28.6% within tertiary B hospitals. Only 36.6% adopted a wide variety of flexible procedures for surging their beds capacity, through early discharge of patients (85.4%), cancellation of elective admissions (63.4%), and transfer patients to primary health care and other facilities (61.0%). However, these hospitals can only surge 12.52% of their total beds within an average of 24 hours (3.76% used extra space, 8.76% could empty beds). Notably, of the14 tertiary B hospitals the surge capacity was even less, as claimed they could only surge 2.48% of their total beds within 24 hours. More than half (61.0%) had mass-casualty triage procedures for admission of patients who require urgent medical care during disasters, and 92% were tertiary A hospitals. Only a few (12.2%) could surge staff using a wide variety of flexible strategies, including recalling all the off work staff back to work (100%), rehiring retired staff (73.2%), suppling living places for staff (61.0%), training and transferring non-critical care staff to support critical care (41.5%), sharing staff from other hospitals (31.7%), and using volunteers or temporary employers (19.5%).

Disaster trainings and drills can be used regularly to improve hospital medical response efficiency. Most of the responding hospitals (95.1%) had disaster training programs and 53.7% had drills, to treat the following emergency types respectively, including: infectious disease (73.2%, 73.2%); mass casualty incidents (53.7%, 48.8%), career poising and food poising (48.8%, 36.6%), and bio-terrorism and nuclear terrorism (14.6%, 7.3%). About 85.4% had training curriculums, and 90.2% updated them regularly. The content of training included cardiopulmonary resuscitation (97.3%), trachea cannulation (89.2%), basic skills for the treatment of trauma (63.6%), transfer of casualties (56.4%), disaster management (12.2%) and triage (12.2%). Approximately one-fifth (19.5%) had attended community drills cooperating with the other emergency facilities. Among them, 25.9% within tertiary A hospitals and only 7.1% within tertiary B hospitals. All the hospitals that conducted such interagency drills were those that had been assigned rescue missions, and they accounted for 61.5% of the mission assigned hospitals.

## Discussion

This study has examined the utility of a comprehensive evaluation framework and its derived questionnaire for furthering the understanding and measurement of the component parts of effective hospital resilience. There are 8 key domains of hospital resilience, and as a result four key factors were extracted from them. Among these factors, emergency medical care is the most important capability, while others (hospital safety, management mechanism, and disaster resources) are supporting capability to guarantee its continuity and surging. This framework of hospital resilience provides a starting point for integrating these key components of hospital resilience together into a comprehensive disaster management framework (including prevention, preparedness, responsiveness, and recovery and adaptation phases). It also seeks to make an achievable goal of improving hospital pre-disaster strength (robustness) and promoting rapidity of response and recovery. This goal can be achieved through a wide range of management approaches including redundancy of processes and resources, and resourcefulness (or flexibility) of plans or strategies (i.e.: can be reflected by some key variables in the survey) [22-26].

Considerable variability in the scope of disaster resilience arrangement of hospitals in the Province was identified through a survey conducted using the self-report questionnaire. We have stratified our analyses by different level of hospitals. It was noticed that in some key areas (e.g., safety evaluation, planning and cooperation, MOUs, personal protective equipment, rescue, surge capacity and drills), there was a difference in disaster resilience arrangements between tertiary A and tertiary B hospitals. This may be due to different levels or types of hospitals having divergent functions in disasters. For example, most (92.3%) of the hospitals that have been assigned missions were tertiary A, and thus they should be more resilient to disasters for health service supply, and should have better arrangements in the above areas for disaster preparedness and response.

This paper offered a four-factor structure as a way of modelling the overall level of hospital resilience and the level of each factor independently. Thus the questionnaire can be used to provide a helpful and comprehensive instrument for assisting hospitals to assess their level of resilience at a regional or a district level in regard to disasters, and assist them in identifying areas for further strengthening their resilience capability through comparison with similar components of other hospitals. The evaluation framework and its key measures in the questionnaire may inform the development of hospital resilience evaluation in other countries.

Similar indicators in this study can be compared with other studies, especially on hospital disaster preparedness and management [13,16]. One survey has been conducted in 2005 to evaluate secondary and tertiary hospitals of Shandong [13]. Comparing its results, it was found that the percentage of most similar indicators in our study is reasonable higher, such as the percentages of: syndromic surveillance systems, single-hazard disaster plans, public and mass media communication protocol, stockpiles of emergency resources, and training programs and drills. Thus, to some extent, it was validated the representativeness of the sample in this study to reflect the status of the province. Additionally, it was found that hospital disaster preparedness in Shandong province is close to Beijing (the capital city), and it is above the average level of preparedness in China, due to economic factors and other factors [13]. It is expected that hospital disaster ability in many other parts of China may lag well behind that of Shandong province. Thus, understanding the status of hospital disaster resilience in this province can be used as the first step in planning effective hospital resilience.

After the SARS crisis, the preparedness of hospitals in China especially for infectious diseases has improved significantly [14]. Our survey revealed that these tertiary hospitals had devised disaster plans and command structures. Almost all of the surveyed hospitals possessed strategies to evacuate and protect existing patients when there is risk in hospitals. Most of them had syndromic surveillance systems. Many had different personal protective equipment and had relevant training programs. A large percentage of them had stockpiles of emergency drugs and resources and had the ability to accept more than 30 cases of infectious diseases within a short period.

These results also highlighted the following shortfall areas in current hospital disaster resilience in Shandong. Firstly, for disaster management mechanism, in US, nearly 67.9% had specific plans for all the essential individual hazards in 2008 [27]. Comparatively, disaster plans in this Province of China had less considerable scope for improving their preparedness for natural disasters, biological, nuclear radiation and other terrorist attacks.

Secondly, for disaster resources, simply stockpiling materials fails to achieve adequate hospital surge capacity, especially in the aftermath of a catastrophic disaster. The community should have functional inter-hospital arrangements to share personnel and resources [4]. In the US, nearly 87.8% of hospitals had MOUs with other hospitals to transfer general patients, 84.1% had contract with other agencies to share suppliers, and more than 70% of hospitals performed mass casualty drills with outside organizations [27]. However, in the Province of China, less than half of the responding hospitals had signed contracts with drug-supplies, and less than one third had signed MOUs with other hospitals to share resources and staff. Also less than half had attended the local coordinating meeting, and only one fifth had attended community-wide drills. The lack of cross-institutional interaction and coordination would likely hinder the availability of resources in a community, and limit timely disaster response.

Finally, continuity of medical care is amongst the most important objectives for prompt and effective response to emergencies. As the experience from developed courtiers, on-site rescue can be enhanced either through dispatched rescue teams (be equipped with living supplies for 3 to 5 days and portable medical equipment) and advanced ‘portable hospitals’ (be equipped with various functional vehicles that can be used for operating surgery, accepting patients, on-site command and communication and etc.) [28]. However, there is still insufficiency of on-site medical rescue, especially a lack of “portability” of critical care service (i.e., patient transport and bringing care to the patient). These two models of on-site rescue still need to be further developed, as they are scalable, mobile and can surge medical care service significantly even after catastrophic disasters [28]. Additionally, medical care capability requires significant surge capacity during disasters, with a critical feature of hospital staffed beds [4]. In US, most hospitals had plans and flexible procedures for surging staffed beds [27]. Also it has been surveyed that in the hospitals of Kentucky, the surge capacity equal to 27% of licensed beds [29]. However, in this study, only less than one fourth of responding hospitals had a wide variety of flexible procedures for surging their beds and emergency staff. The surge capacity within 24 hours is 12.52% of fixed beds, which is relatively low.

Cohesive approaches have been identified using the evaluation framework and its key variables. They can be used by hospital managers and health authorities to enhance general practices to achieve effective disaster resilient. It also can used to assess hospitals, so as to identify the vulnerabilities and improve disaster capability further. These approaches include:

•Hospital safety: (1) Evaluation of locally prioritized hazards, and enforcement of safety standards that need to meet or exceed the local standards; [30,31] (2) Evacuation plan in place and have special procedures to protect and evacuate vulnerable people when there is risk within the hospital.

•Disaster mechanism: (3) The existence of disaster plans that have been developed in advance of a disaster, taking into consideration the communities’ resources, hazards and other unique factors; [2,13] (4) The establishment of a specific department to take responsibility of incident command and control, crisis communication and cooperation, and after-event recovery; [13] (5) Incorporation of the hospital into the overall local disaster planning, including inter-facility cooperation and alternative plans to transfer patients to other hospitals if the hospital is partly destroyed or become unusable [28];

•Disaster resources: (6) Stockpile of self-sufficient resources and emergency drugs for at least 48 to 72 hours, so as to cope with major disasters initially; [32] (7) Establishment of MOUs with other hospitals for transferring patient and the sharing of staffing, equipment, and supplies; [4] A community-wide, integrated, inter-agency network should be built, with local hospitals working together to surge overall capacity collectively [33].

•Emergency medical care: (8) Transportation of the medical staff or transferring patients to hospitals in a timely manner, and the provision of medical care service on site, which can be in the form of rescue teams or ‘portable hospital’ especially during catastrophic disasters; [28] (9) Disaster surge planning should be devised in advance by adoption of a wide variety of flexible strategies (e.g., disaster triage, ability to surge beds and staff, ability to transfer patients, early discharge of patients) for surging medical demands from wide-spread infectious diseases or mass casualty incidents; [27,33,34] (10) Development of hospital internal conditions (e.g., space, beds, treatment protocols and on-call specialists) for treating patients according to type and magnitude of event(s); [13] (11) Systematic and ongoing training and drill staff for emergency medical care skills, equipment usage and disaster management skills in high risk communities [35].

The current study has several limitations. Firstly, the likelihood of non-response bias was likely to exist. As a relatively larger percentage of tertiary A hospitals replied to the survey than the tertiary B and tertiary C hospitals. Although two reminders were sent to the hospital coordinators, there were still 9 hospitals who failed to attend the survey. The follow-up telephones demonstrated that they could not assign the responsible staff to fill the survey, or they are lack of relevant data. Thus it is very possible that the 41 participating hospitals may have relatively good backgrounds of disaster rescue than other 9 nonparticipation hospitals. Also, we suspect that the participating hospitals are better prepared in terms of disaster management than the non-participating hospitals. Secondly, due to the sample size (n = 41), it was possible that not all the significant difference of the mean score of each domain was tested statistically between different hospital categories (as illustrated in Table 1). Despite the limitation, the sample accounted for 52.1% of total hospitals that the study targeted, and did get a response rate over 80%. Thirdly, as the findings are self-reported by the respondents there may be a bias in their reporting. While the inclusion of official documents from Provincial Health Bureaus may have encouraged respondents to complete survey, this may have also been interpreted as an official assessment, thus leading some hospital representatives to overestimate their capability. Fourthly, due to ethical issues, the surveyed hospitals have to be anonymous which impedes comparison with their actual levels of preparedness. Finally, the study was undertaken only in one Province of China. And due to the limitation of funding and investigation time, stratified sampling was used in this study rather than investigating all the tertiary hospitals.

## Conclusions

This study has identified considerable variability in the scope of hospitals’ disaster resilience arrangements in Shandong China. The difference between tertiary A and tertiary B hospitals was also identified in essential areas. A framework was presented which may assist hospitals to better understand what constitutes effective hospital resilience. The framework may also assist hospitals to undertake a self-analysis or audit of their current plans and capacity, and to use this information for future planning. Clearly, more progress is still needed to improve hospital disaster resilience, especially the focus of community-wide disaster cooperation, on-site medical rescue, and hospital patient care surging capacity. It has been shown that hospitals need to take a more cohesive approach to be resilient in order to position themselves to be able to best cope with a potential disaster.

## Competing interests

The authors declare that they have no competing interests.

## Authors’ contributions

SZ and GF designed the study and developed the questionnaire. SZ, LW, LZX and YLZ supervised the data collection and data entry process. SZ performed data checkup, data analysis and drafted the manuscript. All authors participated in writing, revision and approval of the final manuscript.

## Pre-publication history

The pre-publication history for this paper can be accessed here:

http://www.biomedcentral.com/1472-6963/14/135/prepub

## Supplementary Material

Additional file 1 Framework of evaluating hospital disaster resilience. Click here for file

Additional file 2 Questionnaire (English translation version). Click here for file
