# Supplementary material for: Disaster resilience in tertiary hospitals: a cross-sectional survey in Shandong Province, China
Source: BMC Health Serv Res. 2014 Mar 25;14:135. doi: 10.1186/1472-6963-14-135 (PMC3987831; doi:10.1186/1472-6963-14-135)
Supplement: Additional file 1 — Framework of evaluating hospital disaster resilience. [file 1472-6963-14-135-S1.docx]

***Additional file 1: Framework of evaluating hospital disaster resilience***

Framework of evaluating hospital resilience after Modified-Delphi consensus ^[^[^1-11^](#_ENREF_1)^]^

| **Domains** |  | **Sub-domains** | **Measurable indicators** |
| --- | --- | --- | --- |
| Hospital safety^[^[^9-11^](#_ENREF_9)^]^ |  | 1. Surveillance ^[^[^1-9^](#_ENREF_1)^]^ | 1.1 The surveillance events (e.g., abnormity in admission diagnosis, surveillance of emergency room patients and death with unknown causes)  1.2 Analysis, report and share of surveillance information |
|  |  | 2. Hospital infrastructural safety and vulnerability ^[^[^9-11^](#_ENREF_9)^]^ | 2.1 Evaluation of hospital risks and vulnerabilities (e.g., Hospital vulnerability assessment, risks assessment, the strategy to evacuate and protect existing patients)  2.2 The safety standard for hospital critical infrastructures to meet of high risks (e.g., for earthquake, flood, fire and isolation for infectious diseases)  2.3 The alternative emergency energy and facilities for backup (e.g., power, water, oxygen and telecommunication) |
| Disaster leadership and cooperation ^[^[^1-9^](#_ENREF_1)^]^ |  | 3. Leadership ^[^[^1-9^](#_ENREF_1)^]^ | 3.1 Committee staff, workplace |
|  |  | 4. Disaster cooperation ^[^[^1-9^](#_ENREF_1)^]^ | 4.1 The crisis cooperation within hospital  4.2 The cooperation with community facilities (e.g., other hospital facilities, government offices, media, and police, fire department, and other public utilities) |
| Disaster plans^[^[^1-9^](#_ENREF_1)^]^ |  | 5. Plan system ^[^[^1-9^](#_ENREF_1)^]^ | 5.1 Plans for different kinds of disasters (for different single risk)  5.2 The staff coverage of disaster plans within hospital  5.3 The period of evaluating and revising the plan |
|  |  | 6. Operating procedures to execute the plan ^[^[^1^](#_ENREF_1)^]^ | 6.1 The plan initiation (e.g., The rapidity for staff, equipment can be in place when initiating the plan)  6.2 The extent the plan can be execute  6.3 Different responsive procedures for different disaster levels and phases |
| Disaster stockpiles and logistics management^[^[^1-9^](#_ENREF_1)^]^ |  | 7.Disaster resources ^[^[^1-9^](#_ENREF_1)^]^ | 7.1 The stock quantity and types for different emergency resources (e.g., clean water, food, blood, emergency medical suppliers, portable medical equipment )  7.2 The strategies for management of emergency resources(e.g. logistics and distribution, contracts with suppliers and other hospitals, adjusted standards for their usage) |
|  |  | 8. Emergency medication ^[^[^1^](#_ENREF_1)^,^ [^3^](#_ENREF_3)^,^ [^7^](#_ENREF_7)^,^ [^9^](#_ENREF_9)^]^ | 8.1 The stock quantity and types for essential medications for various disasters  8.2 The strategies for management of medications (e.g., drug-distribution plans, drug management policy) |
| Emergency staff ^[^[^1^](#_ENREF_1)^,^ [^5^](#_ENREF_5)^,^ [^6^](#_ENREF_6)^,^ [^9^](#_ENREF_9)^]^ |  | 9. Constitution of emergency group ^[^[^1^](#_ENREF_1)^,^ [^9^](#_ENREF_9)^]^ | 9.1 Staff constitution of emergency expert group (e.g., quantity, specialty, and title)  9.2 Staff constitution of emergency expatriate team (e.g., quantity, specialty, and title) |
|  |  | 10. Staff management ^[^[^9^](#_ENREF_9)^]^ | 10.1 Staff protection and incentives (e.g., insurance, immunization, psychosocial support) |
| Emergency trainings and drills^[^[^1-4^](#_ENREF_1)^,^ [^6^](#_ENREF_6)^]^ |  | 1. 11. Emergency trainings ^[^[^1-4^](#_ENREF_1)^]^ | 11.1 Different incident types for trainings  11.2 The percentage of staff for training  11.3 The contents of trainings (e.g., triage, emergency medical treatment，disaster management knowledge)  11.4 The frequency of trainings |
|  |  | 1. 12. Emergency drill ^[^[^1^](#_ENREF_1)^,^ [^2^](#_ENREF_2)^,^ [^4^](#_ENREF_4)^,^ [^6^](#_ENREF_6)^]^ | 12.1 Different incident types for drills  12.2 The methods for implementing drills (e.g., desktop drill, community-wide drill)  12.3 The frequency of drills |
| Emergency critical care capacity ^[^[^1^](#_ENREF_1)^,^ [^4^](#_ENREF_4)^,^ [^6^](#_ENREF_6)^,^ [^9^](#_ENREF_9)^]^ |  | 1. 13. Disaster surge capacity ^[^[^1^](#_ENREF_1)^,^ [^4^](#_ENREF_4)^,^ [^9^](#_ENREF_9)^]^ | 13.1 Surge capacity of emergency space (The surge rapidity, proportion and strategies for emergency space, emergency beds)  13.2 Surge capacity of emergency resources (The surge rapidity, proportion and strategies for emergency equipment, medication and resource)  13.3 Surge capacity of emergency staff (The surge rapidity, proportion and strategies for emergency staff) |
|  |  | 14. Disaster response procedures ^[^[^6^](#_ENREF_6)^,^ [^9^](#_ENREF_9)^]^ | 14.1 Hospital internal rapid assessment(e.g., evaluate the loss of manpower, beds, equipment after disasters)  14.2 Hospital mass-casualty triage protocol  14.3 The procedures to identify, prioritize, and maintain essential functions(e.g., cancellation of elective admissions, early discharge of patients, making new medical quality standard during disasters, extra protection for vulnerable population) |
|  |  | 15. On-site rescue ^[^[^1^](#_ENREF_1)^,^ [^6^](#_ENREF_6)^]^ | 15.1 The quantity and types of equipment for on-site rescue  15.2 Equipment for referral and counter-referral of special patients  15.3 Communication equipment for on-site rescue |
|  |  | 16. Hospital treatment ^[^[^1^](#_ENREF_1)^]^ | 16.1 Emergency medical treatment place and conditions  16.2 The types and quantity of hospital emergency medical treatment equipment  16.3 Equipment for different types of diseases |
| Recovery mechanism ^[^[^9^](#_ENREF_9)^]^ |  | 17. Recovery and report ^[^[^9^](#_ENREF_9)^]^ | 17.1 Hospital reconstruction and recovery mechanism  17.2 The strategies for community recovery  17.3 The evaluation report |

References:

1. Li X, Huang J, Zhang H**.** An analysis of hospital preparedness capacity for public health emergency in four regions of China: Beijing, Shandong, Guangxi, and Hainan. *BMC Public Health*. 2008;8(1):319.

2. Braun BI, Wineman NV, Finn NL, et al. Integrating hospitals into community emergency preparedness planning. *Ann Intern Med*. 2006;144(11):799-811.

3. Braun BI, Darcy L, Divi C, et al. Hospital bioterrorism preparedness linkages with the community: improvements over time. *Am J Infect Control*. 2004;32(6):317-326.

4. Kaji AH, Lewis RJ**.** Hospital disaster preparedness in Los Angeles county. *Acad Emerg Med*. 2006;13(11):1198-1203.

5. Kollek D, Cwinn AA**.** Hospital emergency readiness overview study. *Prehosp Disaster Med*. 2011;1(1):1-7.

6. Niska RW, Shimizu IM**.** Hospital Preparedness for Emergency Response: United States, 2008. Natl Health Stat Report; 2011:1-14.

7. Higgins W, Wainright C, Lu N, et al. Assessing hospital preparedness using an instrument based on the Mass Casualty Disaster Plan Checklist: results of a statewide survey. *Am J Infect Control*. 2004;32(6):327-332.

8. Thome CD, Levitin H, Oliver M, et al. A pilot assessment of hospital preparedness for bioterrorism events. *Hospital*. 2006;200(299):300.

9. World Health Organization**.** Hospital emergency response checklist-An all-hazards tool for hospital administrators and emergency managemers. The Regional Office for Europe of the WHO; 2011.

10. Pan American Health Organization, WHO**.** Hospital safety index: evaluation forms for safe hospitals. Washington DC: PAHO; 2008.

11. World Health Organization**.** Safe hospitals in emergencies and disasters. World Health Organization (WHO) Regional Office for the Western Pacific; 2010.
