# Supplementary material for: Disaster resilience in tertiary hospitals: a cross-sectional survey in Shandong Province, China
Source: BMC Health Serv Res. 2014 Mar 25;14:135. doi: 10.1186/1472-6963-14-135 (PMC3987831; doi:10.1186/1472-6963-14-135)
Supplement: Additional file 2 — Questionnaire (English translation version). [file 1472-6963-14-135-S2.doc]

***Additional file 2: Questionnaire (English translation version)***

**Questionnaire for assessment of disaster resilience capability**

**in tertiary hospitals of Shandong Province**

**Introduction note (please read first before filling the form)**

1. Public emergencies and disasters in the questionnaire refer to events that suddenly happened and can cause serious impact to the society, which require emergency measures to be taken. These events include natural disasters (e.g., earthquakes, floods), disasters arising from accidents (e.g., transportation incidents, environmental pollution), public health incidents (e.g., emerging infectious diseases, food poisoning) and public security incidents (e.g., terrorism).

2. Fill method: There are two types of questions: (1) choice questions: Unless it is marked with "this question has multiple choices", these questions are single choice (i.e. select one answer), please choose the option that can meet the hospital’s situation, which is after each question (e.g., ①②③) / by tick "√"; (2) fill in the blank: please fill out the relevant data / content into the underscored place after each question. Note the logical question to jump to the next question.

3. Please be sure to complete the form within the required time. After its completion, please report them simultaneously both via e-mail and postal.

**Thank you for your assistance and support to the investigation!**

Hospital name： fill date： month date 2012

The informant（signature） Audit dean（signature） Official seal：（stamp）

**A、The general situation relevant to emergency medicine**

1. Hospital level: Tertiary (e.g., A, B, C).

2. Hospital location（in details to the county, city, and district）:

3. Hospital attribution: ①government health administrative office ②industry hospital ③others

4. Hospital type: ①general hospital ②specialty hospital ③others

5. Hospital mission: ①be assigned regional disaster rescue mission ②not-assigned the mission

**B、Hospital safety**

6. Whether the hospital establishes a syndromic surveillance and early warning system for public health emergencies？①Yes ②No；

6.1 The syndromic that need to be surveillance, report and early warning include:

6.2 Whether develop and require that physicians on duty report any suspicious cases to the hospitals’ presidents? ①Yes ②No;

7. Whether the hospital has direct online reporting system of surveillance information and suspicious symptoms? ①Yes ②No;

7.1 Whether the hospital could analyze surveillance data regularly and share this system with the local health authority? ①Yes ②No;

8. Is there any evaluation of types and impact of the potential risks to hospitals in its location? ①Yes ②No;

8.1 When the disaster occurred, within the hospital is there any hazards identification system for different types of risks? ①Yes ②No;

8.2 When there is hospital internal risk, are there any strategies for hospitals to evacuate and protect existing patients? ①Yes ②No;

9. Is there any evaluation of the safety standards of hospital’s critical infrastructures? (e.g., construction safety standards, safety level of resistance to earthquakes, fires and floods). ①Yes ②No;

9.1 If yes, were the critical infrastructures built to meet or excess the local criteria of resistance to earthquake? ①Yes ②No;

9.2 If yes, were the critical infrastructures built in a higher position in the area to prevent floods? ①Yes ②No;

9.3 If yes, was the critical medical equipment located in a higher level of the building to prevent floods? ①Yes ②No;

9.4. If yes, is there any consideration of the safety standard for the risk of fire? ①Yes ②No;

9.5 If yes, is there any consideration of using isolated pathways and designated areas for infectious diseases within the hospital? ①Yes ②No;

10. When disaster occurred, are there any alternative emergency energy and facilities for backup (including power, water, oxygen and telecommunication)? ①Yes ②No;

**C、 Hospital disaster leadership and cooperation**

11. Is there any disaster committee or disaster group within hospital that is responsible for public emergencies? ①Yes ②No;

11.1 Is there any official document that has been used to establish hospital disaster committee or disaster group? ①Yes ②No;

12. Is there any department within the hospital that has been assigned responsibility for the work relevant to emergencies? ①Yes ②No;

12.1 Is there any official document that has been used to assign emergency relevant work to this department? ①Yes ②No;

13. Is there any coordinating meeting with key staff from different hospital departments during emergencies? ①Yes ②No;

14. Is there any public and mass media communication protocol that can be used for communication during emergencies? ①Yes ②No;

15. Has the hospital attended regional coordinating meeting with other emergency departments during emergencies, such as CDC (Center for Disease Prevention and Control), pre-hospital emergency system, healthcare facilities, blood and resource center, and local government? ①Yes ②No;

**D、 Hospital disaster plan**

16. Is there any general disaster plan and relevant document in place for preparedness of public emergencies? ①Yes ②No;

16.1 Please illustrate the document name of the disaster plan?

17. Are there any specific disaster plans based on the specific requirements of a single hazard, such as infectious diseases, internal medical accidents, public health emergencies, natural disasters, bio-terrorism and nuclear terrorism, and others? ①Yes ②No;

17.1 Please illustrate the document name of the specific disaster plans?

18. Is there any protocol to initiate the plan, so as to guarantee the hospital be in place to face emergencies immediately, (i.e., guarantee staff, equipment and resources are in place immediately)? ①Yes ②No;

19. From the experience of the hospital dealing with the past public emergencies (i.e., mass casualty incident, disasters, pandemics), whether the hospital could operate in accordance with the disaster plan during emergencies? ①Yes ②No;

20. Is there any classification response system to cope with different levels and different phases of events? ①Yes ②No;

21. Is there any evaluation and revision of the disaster plans in the last two years?

①Yes ②No;

22. Is there any dissemination of the content of disaster plans to the key staff (e.g., through regular meetings or training)? ①Yes ②No;

**E、Emergency stockpiles and logistics management**

23. Are there any stockpiles of various types of emergency drugs within hospitals? ①Yes ②No;

23.1 If yes, please illustrate the type names and quantities of these stockpiled drugs?

24. Are there any stockpiles of various types of emergency materials (e.g., food, water, stretcher, and tourniquet)? ①Yes ②No;

24.1 If yes, please illustrate the type names and quantities of the stockpiled materials?

24.2 Whether the hospital has the following personal protective equipment (PPE) *(multi-choices)*?

①biohazard protective suits; ②goggles; ③ventilator; ④N95 Masks

25. When there is mass casualty incident, whether the hospital could be able to load and deliver emergency drugs for on-site rescue? ①Yes ②No;

25.1 If yes, please illustrate the names and quantities of the emergency drugs that could be loaded and delivered for on-site rescue?

26. Whether the hospital has the following strategies for management of drugs and materials?

| **Strategies for management of drugs and materials？** | **Yes** | **No** |
| --- | --- | --- |
| 26.1 Drug-distribution plans to identify distribution priority of drugs during crisis | □ | □ |
| 26.2 Signed contracts with emergency drug-supplies to provide drugs during emergencies | □ | □ |
| 26.3 Signed Memorandum of Understandings (MOUs) with other hospitals to share emergency drugs during emergencies | □ | □ |
| 26.4 Share and obtain these materials from relevant industries during emergencies | □ | □ |
| 26.5 Share and obtain these materials from other hospitals during emergencies | □ | □ |
| 26.6 Others: (please illustrate) | □ | □ |

**F、Emergency Staff**

27. The constitute (i.e., specialty and numbers) of hospital experts group (refer to those members within the hospital that are involved in development of the emergency plans and specific emergency medical treatment)

① General surgical treatment persons, including senior persons;
② General medical treatment persons, including senior persons;
③ Neurosurgery persons, including senior persons;
④ Bone surgery persons, including senior persons;
⑤ Burn persons, including senior persons;
⑥ Psychological persons, including senior persons; ;
⑦ Emergency Department persons, including senior persons;

⑧ ICU persons, including senior persons;
⑨ Nosocomial infections persons, including senior persons;

⑩ Total experts persons, including senior persons;

28. Is there any emergency staff that could be dispatched during disasters for the on-site rescue? ①Yes ②No;

If yes, please fill the constitute (i.e., specialty and numbers) of emergency staff that can be dispatched
① General surgical doctors, persons, general surgical nurse persons ;
② Therapeutic, persons, general medical nurse persons;
③ Neurosurgeon doctors persons, neurosurgery nurses persons;
④ Orthopedic surgeon doctors persons, orthopedic surgeon nurse persons;
⑤ Burn treatment doctors persons, burn treatment nurses persons;
⑥ Psychological doctors persons;
⑦ Emergency department doctors persons, emergency nurses persons;
⑧ ICU doctors persons, ICU nurses persons;
⑨ Infections control doctors persons, infections control nurses persons;
⑩ Manager people persons, including managers persons, information people persons; logistics persons; and other relevant personnel persons

Total doctors persons, total nurses persons;

29. Whether the hospital has the following incentive and protective strategies for management of emergency staff?

| **Incentive and protective strategies for emergency staff?** | **Yes** | **No** |
| --- | --- | --- |
| 29.1 Incentive strategies for emergency staff | □ | □ |
| 29.2 Vaccination for emergency staff and their family members | □ | □ |
| 29.3 Insurance for emergency staff | □ | □ |
| 24.4 Others (please illustrate): | □ | □ |

30. Whether the hospital has the following incentive strategies? (Can be multiple-choices)

① increase the salary; ② increase the vacation; ③ priority for hiring and position promotion; ④ Honors; ⑤ issue the grant

**G、Emergency critical care capability**

***Hospital treatment***

31. The total number of hospital beds sheets;

31.1 Among them, the number of licensed beds sheets;

31.2 The number of beds in emergency department or emergency care center sheets;
31.3 The number of isolation beds sheets;

31.4 The number of orthopedic beds sheets;
31.5 The number of suspension bed for burns sheets;

31.6 The number of emancipated bed for burns sheets;
31.7 The number of surgery rooms ;

31.8 The number of hyperbaric oxygen chambers ;

32. The number of intensive care beds sheets;

32.1 The number of breathing machines ;

32.2 The number of vital signs monitors ;

32.3 The number of defibrillator machines ;

32.4 The number of cardiac resuscitation devices ;

32.5 The number of CRRT devices ;

33. Whether the hospital has capacity (e.g., space, beds and experts) for treating mass casualty of incidents (i.e., here mass casualty treatment capacity refers to each hospital is to assess itself on its capacity to accept at least 30 patients of the same disease within a short period)? ①Yes ②No;

33.1 Whether the hospital has capacity (e.g., space, beds and experts) for treating general mass casualty of trauma? ①Yes ②No;

33.2 Whether the hospital has capacity (e.g., space, beds and experts) for treating mass casualty of infectious diseases? ①Yes ②No;

33.3 Whether the hospital has capacity (e.g., space, beds and experts) for treating mass casualty of blast injury, gunshot wounds and crush injury? ①Yes ②No;

33.4 Whether the hospital has capacity (e.g., space, beds and experts) for treating mass casualty of acute chemical poisoning? ①Yes ②No;

33.5 Whether the hospital has capacity (e.g., space, beds and experts) for treating mass casualty of radiation issues? ①Yes ②No;

***Hospital surge capacity***

34. When disaster occurs, is there any internal evaluation mechanism for rapid assessment of the available emergency resources and the disaster losses? (i.e., manpower, equipment, number of emergency beds)? ①Yes ②No;

35. Are there any prepared spaces and conditions (e.g., electricity, oxygen, water, heat) in place to temporary surge numbers of emergency beds? ①Yes ②No;

35.1 If yes, are there any plans and work procedures for surging emergency beds?

①Yes ②No;

35.2 The maximum surge capacity of emergency beds are sheets (within 24 hours)

36. Are there any procedures and strategies to vacate part of the occupied emergency beds for treating the sick and wounded from emergency events according to the requirement? ①Yes ②No;

36.1 When emergency occurs, according to the instruction from health administrative departments and the actual situation of admitted patients, within 24 hours, the hospital can maximum vacate the occupied emergency beds of sheets?

37. Whether the hospital has a wide variety of flexible procedures for surging beds capacity when it faces the emergencies?

| **Surge procedures for emergency beds** | **Yes** | **No** |
| --- | --- | --- |
| 37.1 cancellation of elective admissions | □ | □ |
| 37.2 early discharge of patients | □ | □ |
| 37.3 transfer patients to primary health care and other facilities | □ | □ |
| 37.4 others (please illustrate)： | □ | □ |

38. Whether the hospital has a wide variety of flexible procedures for surging emergency staff capacity when it faces the emergencies?

| **Surge procedures for emergency staff** | **Yes** | **No** |
| --- | --- | --- |
| 38.1 training and transferring non-critical care staff to support critical care | □ | □ |
| 38.2 recalling all the off-work staff back to work | □ | □ |
| 38.3 rehiring retired staff | □ | □ |
| 38.4 sharing staff from other hospitals | □ | □ |
| 38.5 using volunteers or temporary employers | □ | □ |
| 38.6 suppling living places for staff |  |  |
| 38.7 others (please illustrate)： | □ | □ |

39. Are there any mass-casualty triage procedures for admission of patients who require urgent critical care during disasters? ①Yes ②No;

***On-site Rescue***

40. Whether the hospital has its own ambulances? ①Yes ②No;

40.1 If yes, are there any ward-type ambulances? ①Yes ②No;

40.2 If yes, are there any [negative pressure isolation ambulance](http://www.google.com.au/url?q=http://www.dictall.com/indu/307/30645957454.htm&sa=U&ei=BfgeUtqWCMqaiQeU64GQDA&ved=0CBsQFjAA&sig2=w7V_ijFaHK6xhSyDqPQ1iQ&usg=AFQjCNEI_PUc9GUB3s00O_jyBL9ukvwNrg)s? ①Yes ②No;

40.3 Whether the hospital has on-site command vehicle? ①Yes ②No;

41. Whether the hospital has rescue helicopters and access to a helicopter landing pad?

①Yes ②No;

42. Is there any on-site communication equipment for data transmission, video-audio connection, and remote consultation? ①Yes ②No;

43. Whether the hospital could organise an independent rescue team that is equipped with emergency package of supplies for living 3 days (the teams include those health administrative departments or other departments assigned to construct based on the hospital)?

①Yes ②No;

43.1 If yes, please illustrate the number of staff for the rescue teams, and their departments and specialty:

43.2 If yes, are the rescue teams equipped with portable medical equipment equipped (e.g., portable breathing machine, ECG monitoring machine, and the X-ray machine? ①Yes ②No;

44. Whether the hospital has ‘portable hospital’ or the capability to support field surgery, and other critical care in the field, which is similar to the function of ICU (using vehicles which are equipped with beds and portable medical equipment)? ①Yes ②No;

**H、Emergency training and drills**

45. Are there any disaster or emergency training programs? ①Yes ②No;

46. Are there any disaster or emergency drills? ①Yes ②No;

47. If yes, are there any disaster training programs and drills treating the following emergency types respectively during 2011-2012?

| **Types for disaster training programs and drills** | **has training** | **has drills** |
| --- | --- | --- |
| 47.1 infectious disease | □ | □ |
| 47.2 mass casualty incidents (e.g., natural disasters) | □ | □ |
| 47.3 career poising and food poising | □ | □ |
| 47.4 bio-terrorism and nuclear terrorism | □ | □ |

48. Are there any disaster training curriculums? ①Yes ②No;

48.1 If yes, were the training curriculums updated regularly? ①Yes ②No;

49. In 2011-2012, is there any emergency training including the following content?

| **Content of emergency trainings** | **Yes** | **No** |
| --- | --- | --- |
| 49.1 basic skills for the treatment of trauma | □ | □ |
| 49.2 cardiopulmonary resuscitation | □ | □ |
| 49.3 trachea cannulation | □ | □ |
| 49.4 transfer of casualties | □ | □ |
| 49.5 triage | □ | □ |
| 49.6 disaster management | □ | □ |
| 49.7 others（please illustrate）： | □ | □ |

50. Is there any emergency or disaster training regularly every two years? ①Yes ②No;

51. Are there any disaster drills regularly every two years? ①Yes ②No;

52. Are there any drills the hospital cooperating with all the other emergency facilities of the community? ①Yes ②No;

**I、Recovery and reconstruction**

53. Is there any mechanism of after-event evaluation report? ①Yes ②No;

53.1 If yes, is the following content need to be included in the evaluation report?

| **Evaluation content** | **Yes** | **No** |
| --- | --- | --- |
| 53.1 local high risks re-evaluation | □ | □ |
| 53.2 hospital capability evaluation | □ | □ |
| 53.3 hospital vulnerability evaluation | □ | □ |
| 53.4 experience and lessons that have been learned | □ | □ |
| 53.5 the adaptation strategies in the future | □ | □ |
| 53.6 others (please illustrate)： | □ | □ |

54. Is there any special department that would be assigned to be responsible for the work relevant to recovery and reconstruction? ①Yes ②No;

55. Are there any specific channels of investing money, transferring staff, and purchasing equipment for recovery phases after the event? ①Yes ②No;

56. Whether the hospital has been involved or would be involved in the health related work of the affected communities?

| **Be involved in the health related work of the affected communities?** | **Yes** | **No** |
| --- | --- | --- |
| 56.1 be involved in the design of the recovery strategies for the community | □ | □ |
| 56.2 health evaluation of the community | □ | □ |
| 56.3 health intervention to the community | □ | □ |
| 56.4 rehabilitation for the victims | □ | □ |
| 55.5 psychological consultation for relevant people | □ | □ |
| 55.6 others (please illustrate）： | □ | □ |
